# The Association of Serum Levels of Leptin and Ghrelin with the Dietary Fat Content in Non-Obese Women with Polycystic Ovary Syndrome

**DOI:** 10.3390/nu12092753

**Published:** 2020-09-10

**Authors:** Aleksandra Maria Polak, Anna Krentowska, Agnieszka Łebkowska, Angelika Buczyńska, Marcin Adamski, Edyta Adamska-Patruno, Joanna Fiedorczuk, Adam Jacek Krętowski, Irina Kowalska, Agnieszka Adamska

**Affiliations:** 1 Department of Internal Medicine and Metabolic Diseases, Medical University of Białystok, 15-276 Białystok, Poland; alexandra_1991@op.pl (A.M.P.); a.krentowska@gmail.com (A.K.); a.lebkowska@wp.pl (A.Ł.); irina.kowalska@umb.edu.pl (I.K.); 2 Department of Endocrinology, Diabetology and Internal Medicine, Medical University of Białystok, 15-276 Białystok, Poland; angelika.buczynska@umb.edu.pl (A.B.); akretowski@wp.pl (A.J.K.); 3 Faculty of Computer Science, Bialystok University of Technology, 15-351 Białystok, Poland; m.adamski@pb.edu.pl; 4 Clinical Research Centre, Medical University of Bialystok, 15-276 Białystok, Poland; edyta.adamska@umb.edu.pl (E.A.-P.); j.fiedorczuk@wp.pl (J.F.)

**Keywords:** leptin, ghrelin, macronutrients, PCOS

## Abstract

Women with polycystic ovary syndrome (PCOS) are at an increased risk of developing insulin resistance and abdominal obesity in the state of an improper diet balance. Leptin is a peptide considered to be a satiety hormone that plays an important role in the long-term energy balance, whereas ghrelin is a hormone that controls short-term appetite regulation and is considered a hunger hormone. The aim of the present study was to assess the relationship between serum leptin and ghrelin concentrations and the dietary macronutrient content in PCOS women. We examined 73 subjects: 39 women diagnosed with PCOS by the Rotterdam criteria and 34 healthy controls, matched by the body mass index. The subjects completed a consecutive three-day dietary diary to identify the macronutrient and micronutrient intake. Serum concentrations of leptin and total ghrelin were measured and homeostasis model assessment of insulin resistance (HOMA-IR) was calculated. The studied groups did not differ significantly in terms of the intake of macronutrients (proteins, fats, and carbohydrates) and serum concentrations of ghrelin and leptin (all *p* > 0.05). In the PCOS group, the serum leptin concentration positively correlated with the intake of total fat (r = 0.36, *p* = 0.02), total cholesterol (r = −0.36, *p* = 0.02), saturated fatty acids (r = 0.43, *p* < 0.01), and monounsaturated fatty acids (MUFA) (r = 0.37, *p* = 0.02), whereas the serum ghrelin concentration correlated in an inverse manner with the intake of total fat (r = −0.37, *p* = 0.02), MUFA (r = −0.37, *p* = 0.02), polyunsaturated fatty acids (r = −0.34, *p* = 0.03), and long chain polyunsaturated fatty acids (r = −0.38, *p* = 0.02). In this group, we also found a negative association of HOMA-IR with serum ghrelin levels (r = −0.4, *p* = 0.03) and a positive relationship with the serum leptin concentration (r = 0.5, *p* < 0.01) and relationships between HOMA-IR and total dietary fat (r = 0.38, *p* = 0.03) and MUFA (r = 0.35, *p* = 0.04) intake. In PCOS women, dietary components such as the total fat and type of dietary fat and HOMA-IR are positively connected to serum leptin concentrations and negatively connected to serum ghrelin concentrations, which may influence the energy balance.

## 1. Introduction

Polycystic ovary syndrome (PCOS) is a common endocrinopathy affecting reproductive-age women, with a prevalence reaching 6–20%, depending on the criteria used [1]. Polycystic ovary syndrome is diagnosed by the presence of at least two of the following criteria: oligoovulation and/or anovulation, clinical and/or biochemical hyperandrogenism, and a polycystic ovarian morphology in a transvaginal ultrasound [2]. Insulin resistance and hyperinsulinemia play a crucial role in the pathogenesis of PCOS, which is associated with a higher risk of developing abdominal obesity, metabolic syndrome, pre-diabetes, and, as a consequence, type 2 diabetes, in comparison to the general population [3]. It has been shown that more than 50% of women with PCOS are overweight or obese, which is largely related to dietary patterns [4]. The three principal dietary components (macronutrients) are fats, proteins, and carbohydrates. Fats are classified into subgroups on the basis of the carbon chain length and the degree of saturation [5]. Saturated fatty acids (SFA) have been deemed the predisposing factors of cardiovascular disease [6], while monounsaturated fatty acids (MUFA) and polyunsaturated fatty acids (PUFA) have been considered to be protective factors [7,8]. There are conflicting data on which macronutrient in the diet is connected the most with human obesity [9,10,11,12,13,14]. Some studies have shown an association of an increased consumption of total fat and saturated fatty acids with weight gain and obesity [9,11]. It has been established that fat provides more energy per gram than carbohydrates [12]. Therefore, a higher proportion of fat in the diet can lead to weight gain through an excess energy intake [13]. On the other hand, it has been observed that the percentage of fat in the diet is not connected with excess body fat in Western countries [15]. On the contrary, other authors have demonstrated weight gain with an increased carbohydrate intake [10] and with an increased calorie intake [16]. These conflicting data may result from several methodological issues that make findings from studies on diet and body weight difficult to interpret. Moreover, there are indications that the intake of carbohydrates and fat is more subject to underreporting than the intake of protein, which can affect the results of studies on the macronutrient composition and body weight [12,14] and therefore may confound the association between dietary intake and body weight.

Adipose tissue is considered the primary site able to store energy excess, but also an organ of endocrine secretion. Adipocytes synthesize and secrete biologically active substances, including leptin and ghrelin [17,18]. It has been shown that leptin, which is a product of the leptin gene, is secreted proportional to the amount of adipose tissue [19]. This hormone affects the energy balance, resulting in a decrease in food intake and an increase in energy expenditure [20]. Accordingly, leptin participates in the regulation of metabolism of energy substrates—lipids and carbohydrates [21]. It has been shown that obesity is associated with increased serum leptin concentrations [22,23]. However, in obesity, despite elevated leptin concentrations, the effect of leptin is reduced due to leptin resistance [24,25]. To date, several studies have been published to determine the relationship between dietary components and serum leptin levels, although the existing reports are contradictory. It has been found that high-carbohydrate meals increase serum leptin concentrations in subjects with a normal weight, while obese participants have both fasting and postprandial leptin concentrations higher than those with a normal weight [23]. Pourghassem et al., in a study on PCOS women, showed that high-fat meals reduced the concentrations of circulating leptin [26]. Similar results were obtained by Kong et al. in a study carried out on a group of obese and overweight postmenopausal women. Additionally, they observed an inverse relationship between the serum leptin concentration and the percentage of carbohydrate energy [27]. In contrast to the results of the studies cited above, Yannakoulia et al. showed a positive correlation between the serum leptin concentration and dietary fat intake [28]. In turn, other studies failed to show any association of dietary fat with the serum leptin concentration [29].

Another peptide hormone that has a significant impact on the energy balance, food intake, and regulation of body mass is ghrelin [30]. Ghrelin is secreted by cells in the stomach, pancreas, kidneys, and gonads, and, as mentioned above, by adipose tissue [31]. It plays an important role in the short-term regulation of appetite by stimulating the food intake, and its concentration in blood rises before a meal and decreases after food ingestion [30]. Low serum ghrelin levels were found in conditions of a positive energy balance, such as obesity, and therefore are associated, in an inverse manner, with insulin resistance and type 2 diabetes [32]. Previous studies have shown lower serum ghrelin concentrations in a group of women with PCOS in relation to a control group with a comparable body mass index (BMI) [33]. Studies on the relationship between dietary nutrients and serum ghrelin levels in PCOS subjects are limited and present conflicting results. Pourghassem et al. did not show an association between the serum ghrelin concentration and dietary macronutrient intake in PCOS patients and control subjects [26]. In turn, Barber et al. found that an intake of oral glucose reduced ghrelin secretion in women with PCOS [34].

Some studies have suggested a link between weight gain and imbalance in leptin and ghrelin concentrations in women with PCOS [35,36]; therefore, it seems to be important to determine macronutrients altering leptin and ghrelin concentrations. Accordingly, few studies so far have addressed the relation between dietary macronutrients and serum leptin and ghrelin concentrations in PCOS. Considering the insufficient data and contradictory results of studies conducted in women with PCOS, the purpose of this study was to assess the relationship between serum leptin and ghrelin concentrations and the dietary macronutrient intake in women with PCOS.

## 2. Materials and Methods

### 2.1. Study Participants

The study group consisted of 73 women: 39 subjects with PCOS and 34 healthy women, matched for BMI. Women were recruited from the Department of Endocrinology, Diabetology and Internal Medicine, as well as from the Department of Internal Medicine and Metabolic Diseases, Medical University of Bialystok, Poland. The control group consisted of healthy women recruited from students and staff who met the exclusion criteria and met the following criteria: they did not present hyperandrogenemia or hirsutism; they had a history of regular, ovulatory menstrual cycles; and they had morphologically normal ovaries, assessed by a transvaginal ultrasound. The diagnosis of PCOS was made according to the 2003 Rotterdam European Society of Human Reproduction and Embryology/American Society of Reproductive Medicine (ESHRE/ASRM) PCOS Consensus Workshop Group diagnostic criteria, i.e., the presence of at least two out of three of the following criteria: clinical and/or biochemical hyperandrogenism, oligo/anovulation, and polycystic ovaries on an ultrasound (≥12 follicles measuring 2–9 mm in diameter or an ovarian volume >10 mL in at least one ovary) [2]. The exclusion criteria for all subjects included other causes of irregular menstrual cycles and/or androgen excess, i.e., hyperprolactinemia; Cushing’s syndrome (excluded on the basis of history taking and a physical examination); late-onset congenital adrenal hyperplasia (excluded on the basis of serum levels of 17-hydroxyprogesterone); hypothyroidism or hyperthyroidism; pregnancy (excluded on the basis of an appropriate test) and breastfeeding; type 1 or type 2 diabetes; chronic or acute infection (within the previous 30 days); any other serious medical condition; hormonal contraception and/or anti-androgen therapy (within the previous 6 months); and the use of medications for obesity, hyperglycemia, dyslipidemia, or hypertension.

The study protocol was approved by the Ethics Committee of the Medical University of Białystok, Poland (approval no. APK.002.171.2020) and was concordant with the Declaration of Helsinki. After being fully informed on the purpose and procedures of the study, all subjects signed an informed consent form.

### 2.2. Dietary Intake

Subjects completed a consecutive three-day dietary diary to identify the macronutrient and micronutrient intake. Dietary intake was assessed on the basis of a completed questionnaire regarding the type and amount of products consumed on the previous three days. The subjects were instructed to maintain their lifestyle (including eating behaviors) before the blood collection, and they were asked to record the food intake during the three days preceding the blood collection. The reported amount of studied food items was converted to grams using household measures. For mixed meals, nutrients were calculated based on their components. Food intake data obtained from the participants were analyzed for energy, protein, carbohydrates, total fat, SFA, MUFA, PUFA, long chain polyunsaturated fatty acids (LC-PUFA), total cholesterol, dietary fiber, vitamins, and trace elements, as well as the percentage of energy from protein, carbohydrate, fat, and alcohol. The content of daily nutrient intake was calculated by the Diet 5.0 program developed by the Institute of Food and Nutrition, Poland.

### 2.3. Anthropometric Measurements

All women underwent a physical examination. The body mass index was calculated as the body weight in kilograms divided by the height in meters squared (kg/m^2^). The waist-hip ratio (WHR) was calculated from the waist circumference (the smallest circumference between the rib cage and the iliac crest) and hip circumference (the maximum circumference at the level of the femoral trochanters), measured in the standing position.

### 2.4. Biochemical Analysis

Blood was sampled in the morning between the 3rd and 6th day of the menstrual cycle, or, if the woman suffered from amenorrhea, in any phase of the cycle at least 3 months from the last spontaneous menses. Fasting concentrations of plasma glucose and serum insulin, as well as plasma concentrations of glucose and serum levels of insulin two hours after the ingestion of 75 g of glucose, were assessed. Plasma glucose concentrations were determined with the hexokinase method and serum insulin levels were determined by the immunoradiometric method (DIAsource ImmunoAssays S.A., Louvain-La-Neuve, Belgium) (minimum detectable concentration (MDC)—1 µIU/mL; intra-assay coefficient of variation (CV)—below 2.2%, inter-assay CV—below 6.5%). Plasma total cholesterol (TC), high-density lipoprotein cholesterol (HDL-C), and triglycerides (TG) were assessed by the enzymatic colorimetric method (Cobas c111, Roche Diagnostic Ltd., Rotkreuz, Switzerland). The plasma low-density lipoprotein cholesterol (LDL-C) concentration was calculated with Friedewald’s formula.

Serum leptin concentrations were measured using an immunoenzymatic method (Human Leptin ELISA, BioVendor, Brno, Czech Republic) (MDC—0.2 ng/mL; intra-assay CV—5.9%, inter-assay CV—5.6%). Total and active ghrelin concentrations were measured by the radioimmunometric method, using specific antibodies for the total and active ghrelin form, respectively. Total ghrelin concentrations were assayed using the commercial kit for ghrelin (total) (GHRT-89HK, RIA, Millipore, USA, with MDC—100 pg/mL, intra-assay CV—below 10.0%, inter-assay CV—below 17.8%). The level of the active form of ghrelin was measured by a ghrelin (active) kit (GHRA-88HK, RIA, Millipore, Burlingtone, MA, USA, with MDC—10 pg/mL, intra-assay CV—below 9.5%, inter-assay CV—below 16.2%). Accordingly, the leptin/ghrelin ratio was calculated.

The levels of serum follicle-stimulating hormone (FSH) and luteinizing hormone (LH) were assessed with the immunoradiometric method (DIAsource ImmunoAssays S.A., Louvain-La-Neuve, Belgium) (LH: intra-assay CV—below 3.9%, inter-assay CV—below 8%, FSH: intra-assay CV—below 2%, inter-assay CV—below 4.4%). The serum concentration of estradiol was determined by a radioimmunoassay (DIAsource ImmunoAssays S.A., Louvain-La-Neuve, Belgium) (MDC—2.7 pg/mL, intra-assay and inter-assay CV—4.7% and 10.4%, respectively). The measurement of total testosterone was performed using a radioimmunoassay (DIAsource ImmunoAssays S.A., Louvain-La-Neuve, Belgium) (MDC—0.05 ng/mL, intra-assay CV—3.3%, inter-assay CV—4.8%). Serum sex hormone–binding globulin (SHBG) was measured by an immunoradiometric assay (ZenTech, Angleur, Belgium) (intra-assay CV—below 5.2%, inter-assay CV—below 5.8%). The free androgen index (FAI) was calculated as the serum total testosterone (nmol/L) × 100/SHBG (nmol/L) ratio [37]. The serum TSH concentration was measured with the immunoradiometric method (DIAsource ImmunoAssays S.A., Louvain-La-Neuve, Belgium) (sensitivity 0.025 µIU/mL; intra-assay CV—0.6%; inter-assay CV—2.1%). 

Insulin resistance was estimated by using the homeostasis model assessment index (HOMA-IR), which was calculated according to the following formula: (fasting insulin [µU/mL] × fasting glucose [mmol/L])/22.5 [38]. 

### 2.5. Statistical Analysis

Statistical analyses were performed using the Statistica 13.3 package (Statsoft, Cracow, Poland). The variables were tested for a normal distribution using the Shapiro–Wilk test. Due to a non-normal distribution of the data, non-parametric tests were applied and all values were expressed as the median and interquartile range. Comparisons of the PCOS and control group were performed by the Mann–Whitney U test. The Spearman test was used for correlation analysis. A *p*-value < 0.05 was considered statistically significant. We did not correct for multiple correlation analyses employing the same subjects and involving many parameters.

## 3. Results

The clinical and biochemical characteristics of the studied groups are presented in Table 1. The studied groups did not differ significantly in terms of BMI and WHR (all *p* > 0.05), although women with PCOS were younger than controls. In the PCOS group, the serum level of total testosterone and FAI were significantly higher in comparison to the control group, whereas the serum SHBG concentration was found to be lower in the PCOS group in comparison to the control group (all *p* < 0.05) (Table 1). 

We did not observe significant differences in total ghrelin, active ghrelin, and leptin serum concentrations or the leptin/ghrelin ratio between the groups (all *p* > 0.05) (Table 1). 

The studied groups did not differ in the amount of daily macronutrient (proteins, fats, and carbohydrates), as well as SFA, MUFA, PUFA, LC-PUFA, and micronutrient intake (sodium, potassium, calcium, phosphor, magnesium, iron, zinc, Vitamin A, Vitamin E, Vitamin D, Vitamin C, Vitamin B3, Vitamin B6, Vitamin B12, and iodine) (all *p* > 0.05). Moreover, no differences in the total energy intake during the day were observed between the studied groups (*p* = 0.51). We did not observe differences between the percentage of energy from fat, proteins, and carbohydrates between PCOS women and the control group (all *p* > 0.05) (Table 2).

We observed relationships between the BMI and serum concentration of leptin (r = 0.61, *p* < 0.01) and ghrelin (r = −0.39, *p* = 0.01) in PCOS women. We also noticed a correlation between the BMI and serum concentration of leptin (r = 0.69, *p* < 0.01), but no relationship between the BMI and serum level of ghrelin (r = −0.1, *p* = 0.56) in the control group. 

In the PCOS group, the serum leptin concentration correlated with the total fat (r = 0.36, *p* = 0.02), SFA (r = 0.43, *p* < 0.01), and MUFA (r = 0.37, *p* = 0.02) contained in the diet, whereas serum ghrelin concentrations correlated, in an inverse manner, with the total fat (r = −0.37, *p* = 0.02), total cholesterol (r = −0.36, *p* = 0.02), MUFA (r = −0.37, *p* = 0.02), PUFA (r = −0.34, *p* = 0.03), and LC-PUFA (r = −0.38, *p* = 0.02) contained in the diet. Additionally, in the group of PCOS women, we found a negative correlation between the acylated ghrelin concentration and proteins contained in the diet (r = −0.35, *p* = 0.03). Furthermore, in the PCOS group, we observed a positive association between the leptin/ghrelin ratio and total fat (r = 0.45, *p* < 0.01), SFA (r = 0.49, *p* < 0.01), MUFA (r = 0.45, *p* < 0.01), and PUFA (r = 0.34, *p* = 0.04) contained in the diet. There was also a correlation of borderline significance between the leptin/ghrelin ratio and the percentage of energy from fats (r = 0.32, *p* = 0.05) (Table 3).

In the control group, we did not observe relationships between serum ghrelin concentrations and dietary macronutrients (all *p* > 0.05). We found a correlation between the serum leptin concentration and protein intake in the diet (r = −0.45, *p* < 0.01) and between the leptin/ghrelin ratio and proteins in the diet (r = −0.44, *p* < 0.01) (Table 3).

In the PCOS group, we observed a negative association of HOMA-IR with serum ghrelin levels (r = −0.4, *p* = 0.03) and a positive relationship with the serum leptin concentration (r = 0.5, *p* < 0.01). We also found an association between HOMA-IR and the dietary intake of total fat (r = 0.38, *p* = 0.03) and MUFA (r = 0.35, *p* = 0.04) in this group (Table 4). 

We observed a positive relationship between FAI and the serum levels of leptin (r = 0.38, *p* = 0.01) and a negative association between the serum concentration of SHBG and serum levels of leptin (r = −0.4, *p* < 0.01) in PCOS women (Table 4). In the control group, we did not observe relationships of total testosterone and FAI with serum levels of leptin and ghrelin (all *p* > 0.05). 

Furthermore, in the PCOS group, we observed a positive association between FAI and SFA intake (r = 0.34, *p* = 0.04) and a negative association between SHBG concentrations and the dietary intake of total fat (r = −0.38, *p* = 0.02), SFA (r = −0.51, *p* < 0.01), and MUFA (r = −0.35, *p* = 0.03) (Table 4). In the control group, we did not observe relationships of FAI and SHBG with fat or subgroups of fatty acids (all *p* > 0.05). 

## 4. Discussion

In our study, we did not observe significant differences in diet and serum leptin and ghrelin concentrations between PCOS patients and control subjects. However, we found a positive association of the dietary total fat, SFA, and MUFA intake with serum leptin concentrations exclusively in non-obese PCOS women; whereas, in the control group, we did not notice the above associations. Additionally, we observed a negative relationship between serum levels of leptin and HOMA-IR and a positive association between the dietary fat intake and HOMA-IR. According to the available evidence, dietary SFA increase the risk of obesity [9], which may result in changes in leptin concentrations. Some studies have reported that a high-fat diet is associated with an increased leptin concentration [39,40,41], while other studies have shown that dietary fat reduced the serum leptin concentration [26,42,43]. In turn, some researchers did not show the influence of dietary fat on serum leptin concentrations [44]. The above conflicting data may reflect the lack of adjustment for gender, the total energy intake, and BMI. Yannakoulia et al. [28] found that the free leptin index is negatively associated with the energy intake from carbohydrates and positively associated with the energy intake from dietary fat. Moreover, they observed that the serum leptin concentration reflects the amount of body fat and is higher in women compared with men. Sexual dimorphism of the body fat distribution or differences in sex steroid hormones between genders have been proposed to be responsible for the observed differences in leptin concentrations [28]. It has been found that the intake of SFA increases serum leptin concentrations [45]. This relationship can be explained by the induction of insulin resistance by dietary fat and SFA [46]. The association between dietary fat intake and an increase in the serum leptin concentration could be related to changes in serum insulin concentrations. As mentioned previously, we found a positive association between the dietary fat intake and HOMA-IR and a positive relationship between serum levels of leptin and HOMA-IR. It has been previously reported that serum leptin concentrations are positively associated with insulin resistance in obese women [47], and that an elevated serum insulin concentration that follows insulin resistance can stimulate leptin mRNA expression in adipocytes and increase circulating leptin concentrations [48,49]. Moreover, the available data have shown that the ingestion of a high-fat diet induces a state of leptin resistance in the absence of an increasing serum leptin concentration and body fat mass in rodents [50]. These findings suggest that certain macronutrients may be involved in the induction of leptin resistance prior to an increase in the leptin concentration and body weight and may play an important role in the development of obesity. 

In our study, we did not observe an association between the dietary fiber intake and serum leptin concentration; however, a study carried out on young Japanese women showed that a higher intake of dietary fiber was associated with a lower serum leptin concentration [29]. Additionally, the protein and PUFA intake was inversely related to the serum leptin concentration, although this association was dependent on the intake of other nutrients. The suggested explanation of this pattern is that the dietary fiber intake may decrease the serum leptin concentration directly through a decrease in leptin production or indirectly through an increase in leptin sensitivity, which in turn leads to a decrease in leptin production through feedback mechanisms [29]. 

In our study, we did not observe differences in serum levels of ghrelin between women with PCOS and control subjects, although our results indicate that ghrelin serum concentrations are connected, in an inverse manner, to the dietary intake of total fat, MUFA, PUFA, LC-PUFA, and total cholesterol in the PCOS group. The findings of previously conducted studies suggest that, besides leptin disturbances, an imbalance in the ghrelin concentration may also be associated with weight gain in women with PCOS [35,36,51,52]. Our results contrast with those of some other studies, which showed that an increased intake of fat and carbohydrates is associated with higher ghrelin concentrations [27] and those which showed no relationship between dietary macronutrients and serum ghrelin concentrations [26,53]. The above conflicting data may reflect the differences in ethnic groups; lack of adjustment for potential confounding factors, such as the BMI of the studied group; or different methods of diet analysis. An inverse association between ghrelin concentrations and the dietary intake of fat and fatty acids observed in our study can be explained by the involvement of fat and fatty acids in the induction of insulin resistance, which is suggested by the positive relationship of total fat and MUFA in the diet with HOMA-IR. Moreover, it has been previously shown that an increased insulin concentration leads to a decrease in the serum ghrelin concentration [54,55].

It has been proposed that in the analysis of the hormonal response to meal intake, leptin and ghrelin effects should be combined [56], as the energy balance and the final clinical effect depend on the interplay between both hormones [57]. Therefore, in our study, we also analyzed the ratio of leptin and ghrelin. In the PCOS group, we found a positive correlation between the leptin/ghrelin ratio and total fat, SFA, MUFA, and PUFA contained in the diet. Previous studies noted that a higher leptin/ghrelin ratio was associated with a lower resting metabolic rate [58]. It has been found that overweight and obese subjects present a higher leptin/ghrelin ratio [56]. Therefore, we can suspect that a higher intake of total fat, SFA, MUFA, and PUFA in PCOS individuals may increase the leptin/ghrelin ratio and contribute to the development of obesity.

As previously mentioned, in our study, we did not find any significant differences in leptin and ghrelin concentrations between the PCOS patients and the control group. These results support the previous findings of other studies, which presented similar leptin and ghrelin levels in both PCOS patients and control group [59]. Contradictory results have been reported by Pekhlivanov et al. [60] and Jalilian et al. [61], who noted increased leptin concentrations in PCOS patients. In addition, it has been found, similarly to our study, that the serum leptin concentration is closely related to BMI [61]. Therefore, these controversial results and a lack of differences in the serum leptin concentration shown in our study may result from the fact that the studied groups did not differ in BMI and our groups were non-obese. This statement is based on the fact that leptin is predominantly produced by adipocytes, and therefore, patients with a higher BMI, which reflects the amount of adipose tissue, may present higher serum leptin concentrations [61]. Interestingly, it has been proposed that, in part by increased intra-follicular levels of leptin, obesity directly affects ovarian functions in PCOS and may induce a relative resistance to gonadotropins [62]. In addition, the small size of the group may affect the obtained results, which was indicated in the limitations of this study. Another explanation of the obtained results could be connected to the fact that we only studied Caucasian women, in contrast to other studies. 

In our study, we also observed a negative correlation between the serum acylated ghrelin concentration and diet protein intake in the group of PCOS women. Proteins have been shown to reduce the appetite more than equivalent calories from carbohydrates or lipids [63,64,65]. The higher satiety associated with the protein intake seems to be related to ghrelin suppression [64]. Studies have shown that postprandial ghrelin suppression is greatest after protein ingestion [66,67]. Moreover, protein intake suppresses ghrelin longer than other types of macronutrients [64,68]. The prolonged suppression of ghrelin after the intake of protein might be associated with a prolonged emptying of proteins from the stomach [66]. Moreover, proteins are able to stimulate the secretion of specific gastrointestinal peptides (cholecystokinin, glucagon-like peptide-1, and gastric inhibitory polypeptide), which delay gastric emptying [64,69]. Additionally, after protein intake, the concentration of circulating amino acids increases, which stimulates hepatic gluconeogenesis, preventing hypoglycemia and thus causing satiety [69]. Therefore, based on the inverse relationship between the serum acylated ghrelin concentration and diet protein intake in the PCOS group, we can speculate that the mechanisms described above are sufficient in these women. However, this association requires further research.

We also noticed that PCOS women had higher concentrations of serum testosterone and FAI, whereas SHBG was lowered compared to healthy women. Additionally, in our study, we only observed a relationship between FAI and serum levels of leptin in PCOS women. In the literature, the relationship between the serum concentration of leptin and androgens in PCOS is still controversial, and the interactions between gonadotropins, insulin, and leptin are very complex [35]. Leptin inhibits the insulin-mediated promotion of gonadotropin-stimulated ovarian steroidogenesis [35]. However, in our study, we did not observe differences in the serum concentration of leptin and ghrelin between PCOS women and healthy controls, despite the fact that a high proportion of women with PCOS were hyperandrogenic. In the aforementioned study, it has been shown that an elevated serum concentration of leptin is associated with elevated levels of testosterone in PCOS women. Therefore, it has been postulated that there is as yet an undefined mechanism, probably mediated by insulin, that would explain these relationships [35]. Elevated levels of androgens in PCOS are due to an excessive production of androgens by the ovaries [70] in the state of insulin resistance [71]. Dietary components seem to play a role in the regulation of androgens and SHBG concentrations in PCOS subjects [72,73], and it is possible that insulin resistance is involved in this process. It has been found that diets containing higher amounts of SFA are able to reduce insulin sensitivity more than diets consisting of other types of fatty acids [74]. In our study, we found that SFA correlate positively with FAI and negatively with SHBG. Moreover, the dietary total fat intake was negatively associated with SHBG concentrations. Given the important role of insulin resistance in the development of hyperandrogenemia, it is possible that fat and saturated fatty acids induce insulin resistance and compensatory hyperinsulinemia, which affect the changes in the androgen level.

The major limitation of the present study is that women in the PCOS group were younger than the control individuals, what could have affected the results when comparing the differences in parameters which are partially dependent on age. Another limitation of our study is the relatively small sample size; however, the participants were very well-characterized. Moreover, we only measured leptin and ghrelin concentrations in the fasting state, without an assessment of the postprandial state or hormone response to given macronutrient test meals. The use of a self-reported dietary intake may also be considered a limitation of the present study because it may not accurately reflect the real amount and type of food consumed. In addition, the results indicate a relationship of leptin and ghrelin with dietary macronutrients; however, they do not explain the causal relationships, but only the associations. Considering the results of our study, which suggest an association of dietary macronutrients with serum leptin and ghrelin concentrations, it seems to be important to find the causal relationship arising from these findings. Therefore, future research is needed. In order to reach a full understanding of the mechanism and effect of dietary macronutrients, as well as the possible interactions between dietary components and the energy balance, the omics approach may be considered. This approach would provide more accurate information on mechanisms involved in the response to the consumption of different dietary macronutrients [75]. Omics-based nutrition research can identify those individuals who are likely to respond maximally and will provide personalized dietary recommendations [76,77] for women with PCOS. This is very important for this group of patients, as they have an increased risk of obesity, metabolic syndrome, and type 2 diabetes in relation to the general population [3]. However, this approach requires advanced skills for data analysis and is relatively expensive; therefore, the availability of sufficient funding is the main limiting factor for performing such studies [78]. Additionally, in our study, we included a unique, non-obese group of women with PCOS, compared to their control counterparts. In our study, PCOS women differed from the control group in terms of the serum levels of total testosterone, SHBG, and FAI, whereas we did not observe differences between the studied groups in terms of the serum concentration of glucose and insulin during OGTT, as well as HOMA-IR. However, we used HOMA-IR instead of a euglycemic hyperinsulinemic clamp to estimate insulin resistance and we therefore cannot exclude insulin resistance in the non-obese PCOS group. On the other hand, this group differs profoundly from obese PCOS women. Therefore, it is an advantage of our study that we examined non-obese subjects, because the impact of obesity on the studied parameters was removed. It is important to study non-obese PCOS women, because they could present a tendency for weight gain and obesity [3] in the future. Moreover, it has been shown that women with PCOS are characterized by atherogenic dyslipidemia [79]. In our study, we did not observe differences in serum levels of lipids between non-obese PCOS women and the control group, but we found relationships between fat in the diet and the leptin and ghrelin serum concentration. Therefore, dietary fat could impact the physiological function in PCOS women, and it could be indicative of an abnormal lipid function in PCOS per se. Therefore, it seems to be important to assess whether the non-obese PCOS group has any metabolic abnormalities and what kind of abnormalities they present before the onset of obesity, which could be accompanied by leptin resistance [24]. Based on our findings, we can speculate that limiting food products high in fat (especially SFA) would be beneficial for the appropriate control and management of the metabolic status of PCOS patients. However, to confirm this hypothesis, a prospective study should be performed. 

## 5. Conclusions

In PCOS women, dietary components, especially the total fat intake and the types of dietary fat, as well as HOMA-IR, correlate positively with serum leptin concentrations and negatively with serum ghrelin concentrations, which may influence the energy balance. 

## Figures and Tables

**Table 1:** Clinical and biochemical characteristics of the studied groups.

	Control Group (*n* = 34)	PCOS (*n* = 39)	*p* Value
Age (years)	26 (24.0–28.0)	23 (21–27)	<0.01 *
BMI (kg/m^2^)	22.92 (20.64–24.9)	23.54 (21.47–25.93)	0.24
WHR	0.8 (0.77–0.85)	0.83 (0.79–0.87)	0.12
FSH (IU/L)	5.64 (4.43–6.56)	5.69 (4.39–7.0)	0.62
LH (IU/L)	3.79 (2.93–6.0)	4.05 (2.94–5.56)	0.62
Estradiol (ng/L)	66.68 (48.46–77.96)	58.26 (49.17–76.46)	0.72
TT (ng/mL)	0.63 (0.49–0.79)	0.78 (0.61–0.89)	0.01 *
SHBG (nmol/L)	72.23 (56.51–91.36)	47.02 (31.56–64.68)	<0.01 *
FAI	2.79 (2.01–4.01)	5.03 (2.86–8.5)	<0.01 *
TSH (mIU/L)	1.69 (1.36–2.33)	2.17 (1.34–2.8)	0.25
Glucose 0′ OGTT (mg/dL)	92 (88–99)	93 (89–98)	0.81
Glucose 120′ OGTT (mg/dL)	94 (86–104)	96 (80–106)	0.97
Insulin 0′ OGTT (uIU/mL)	7.68 (6.78–10.16)	9.01 (6.36–11.67)	0.42
Insulin 120′ OGTT (uIU/mL)	34.05 (22.3–45.21)	38.37 (25.3–62.91)	0.15
HOMA-IR	1.81 (1.52–2.31)	2.19 (1.42–2.86)	0.47
Total cholesterol (mg/dL)	170.5 (149–195)	172 (155–180)	0.62
HDL-cholesterol (mg/dL)	73 (60–81)	68 (57–75)	0.11
LDL-cholesterol (mg/dL)	88.9 (72–105)	90.8 (81–103)	0.76
TG (mg/dL)	50.5 (40–70)	57 (47–79)	0.14
Ghrelin (total) (pg/mL)	1017.60 (823.06–1124.05)	869.39 (702.34–1101.45)	0.08
Ghrelin (active) (pg/mL)	39.62 (33.58–51.12)	41.95 (32.53–55.7)	0.78
Leptin (ng/mL)	9.94 (5.59–14.94)	12.84 (5.68–19.75)	0.46
Leptin/Ghrelin ratio	0.01 (0.01–0.02)	0.01 (0.01–0.03)	0.28

Values are expressed as the median (interquartile range): * *p* < 0.05. Abbreviations: BMI: body mass index; WHR: waist-hip ratio; FSH: follicle-stimulating hormone; LH: luteinizing hormone; TT: total testosterone; SHBG: sex hormone-binding globulin; FAI: free androgen index; TSH: thyroid-stimulating hormone; OGTT: oral glucose tolerance test; HOMA-IR: homeostasis model assessment of insulin resistance; TG: triglycerides; HDL: high-density lipoprotein; LDL: low-density lipoprotein; PCOS: polycystic ovary syndrome.

**Table 2:** Macronutrient and micronutrient intake in the studied groups.

	Control Group (*n* = 34)	PCOS (*n* = 39)	*p* Value
Carbohydrate (g)	224.74 (167.37–278.24)	201.01 (166.21–227.37)	0.28
Protein (g)	64.58 (58.22–75.3)	66.34 (59.67–78.49)	0.4
Total fat (g)	51.58 (42.52–63.79)	52.35 (36.91–68.46)	1
SFA (g)	18.40 (15.21–22.59)	18.72 (13.56–23.05)	0.74
MUFA (g)	20.9 (16.2–25.76)	21.89 (14.5–28.59)	0.95
PUFA (g)	7.83 (5.47–10.46)	7.24 (5.52–10.52)	0.8
LC-PUFA (g)	0.06 (0.02)	0.07 (0.03–0.25)	0.55
Total dietary cholesterol (mg)	190.11 (154.66–290.59)	239.19 (154.37–307.62)	0.53
Total dietary fiber (g)	17.96 (12.89–26.33)	16.02 (12.21–22.16)	0.32
Total energy intake (kcal)	1575.20 (1240.07–1935.8)	1556.85 (1217.86–1791.39)	0.51
Percentage of energy from carbohydrate (%)	52.0 (43.51–56.27)	49.55 (43.16–52.52)	0.27
Percentage of energy from protein (%)	16.45 (14.56–19.41)	18.39 (15.28–21.39)	0.13
Percentage of energy from fat (%)	28.26 (24.25–36.04)	30.58 (26.57–35.05)	0.69
Sodium (mg)	2507.58 (2240.78–3177.19)	2454.1 (2161.57–3307.55)	1
Potassium (mg)	2864.78 (2205.7–4234.38)	2725.26 (2304.4–3706.78)	0.47
Calcium (mg)	643.95 (458.71–807.84)	603.75 (418.67–773.5)	0.33
Phosphor (mg)	1120.78 (902.5–1449.26)	1138.16 (958.67–1403.96)	0.87
Magnesium (mg)	274.67 (232.25–330.97)	287.56 (206.36–351.9)	0.85
Iron (mg)	9.23 (7.52–15.68)	9.21 (8.08–12.11)	0.65
Zinc (mg)	8.3 (6.87–9.8)	8.43 (6.89–9.72)	0.89
Vitamin A (µg)	792.2 (614.91–1128.55)	839.89 (537.03–1196.49)	0.63
Vitamin E (mg)	7.43 (4.96–11.42)	7.7 (5.35–9.85)	0.71
Vitamin B3 (mg)	18.57 (12.45–23.04)	17.27 (12.68–22.5)	0.93
Vitamin B6 (mg)	1.62 (1.34–2.26)	1.69 (1.3–2.18)	0.82
Vitamin B12 (mg)	2.44 (2.02–3.93)	2.56 (1.8–4.13)	0.93
Vitamin C (mg)	70.59 (44.93–111.29)	81.3 (60.8–112.08)	0.5
Vitamin D (µg)	2.14 (1.18–3.35)	1.66 (1.32–3.23)	0.87
Iodine (µg)	95.53 (62.49–128.72)	95.99 (69.72–131.17)	0.57

Values are expressed as the median (interquartile range): The level of significance was accepted at *p* < 0.05. Abbreviations: SFA: saturated fatty acids; MUFA: monounsaturated fatty acids; PUFA: polyunsaturated fatty acids; LC-PUFA: long chain polyunsaturated fatty acids; PCOS: polycystic ovary syndrome.

**Table 3:** Correlations of leptin and ghrelin concentrations and the leptin/ghrelin ratio with the dietary intake of macronutrients.

	Control Group (*n* = 34)			PCOS (*n* = 39)		
	Leptin	Ghrelin	Leptin/Ghrelin	Leptin	Ghrelin	Leptin/Ghrelin
Total energy intake (kcal)	r = −0.14	r = 0.23	r = −0.18	r = 0.19	r = −0.2	r = 0.25
	*p* = 0.45	*p* = 0.19	*p* = 0.32	*p* = 0.26	*p* = 0.23	*p* = 0.13
Carbohydrate (g)	r = −0.05	r = 0.34	r = −0.13	r = 0.06	r = −0.01	r = 0.11
	*p* = 0.79	*p* = 0.05	*p* = 0.48	*p* = 0.7	*p* = 0.94	*p* = 0.52
Protein (g)	r = −0.45	r = 0.07	r = −0.44	r = 0.21	r = −0.19	r = 0.2
	*p* < 0.01 *	*p* = 0.7	*p* < 0.01 *	*p* = 0.19	*p* = 0.24	*p* = 0.23
Total fat (g)	r = −0.02	r = 0.01	r = −0.01	r = 0.36	r = −0.37	r = 0.45
	*p* = 0.9	*p* = 0.94	*p* = 0.96	*p* = 0.02 *	*p* = 0.02 *	*p* < 0.01 *
SFA (g)	r = −0.12	r = −0.02	r = −0.09	r = 0.43	r = −0.24	r = 0.49
	*p* = 0.5	*p* = 0.92	*p* = 0.63	*p* < 0.01 *	*p* = 0.13	*p* < 0.01 *
MUFA (g)	r = 0.04	r = −0.02	r = 0.05	r = 0.37	r = −0.37	r = 0.45
	*p* = 0.83	*p* = 0.9	*p* = 0.79	*p* = 0.02 *	*p* = 0.02 *	*p* < 0.01 *
PUFA (g)	r = −0.07	r = −0.07	r = −0.02	r = 0.25	r = −0.34	r = 0.34
	*p* = 0.69	*p* = 0.71	*p* = 0.89	*p* = 0.12	*p* = 0.03 *	*p* = 0.04 *
LC-PUFA (g)	r = −0.06	r = 0.09	r = −0.12	r = 0.1	r = −0.38	r = 0.18
	*p* = 0.75	*p* = 0.59	*p* = 0.5	*p* = 0.56	*p* = 0.02 *	*p* = 0.27
Total dietary cholesterol (mg)	r = −0.09	r = 0.04	r = −0.1	r = 0.13	r = −0.36	r = 0.2
	*p* = 0.62	*p* = 0.82	*p* = 0.56	*p* = 0.45	*p* = 0.02 *	*p* = 0.23
Percentage of energy from carbohydrate (%)	r = 0.12	r = 0.32	r = 0.02	r = −0.11	r = 0.22	r = −0.12
	*p* = 0.48	*p* = 0.07	*p* = 0.91	*p* = 0.52	*p* = 0.17	*p* = 0.46
Percentage of energy from protein (%)	r = −0.35	r = −0.24	r = −0.27	r = −0.05	r = −0.1	r = −0.1
	*p* = 0.04 *	*p* = 0.16	*p* = 0.13	*p* = 0.78	*p* = 0.53	*p* = 0.56
Percentage of energy from fat (%)	r = −0.01	r = −0.24	r = 0.06	r = 0.25	r = −0.28	r = 0.32
	*p* = 0.97	*p* = 0.18	*p* = 0.72	*p* = 0.12	*p* = 0.09	*p* = 0.05

Data are derived from the Spearman correlation coefficient. The level of significance was accepted at * *p* < 0.05. Abbreviations: SFA: saturated fatty acids; MUFA: monounsaturated fatty acids; PUFA: polyunsaturated fatty acids; LC-PUFA: long chain polyunsaturated fatty acids; PCOS: polycystic ovary syndrome.

**Table 4:** Correlations between hormones, HOMA-IR, and the dietary intake of macronutrients in polycystic ovary syndrome (PCOS) women.

PCOS (*n* = 39)			
	HOMA-IR	SHBG	FAI
Leptin (ng/mL)	r = 0.5 *p* < 0.01 *	r = −0.4 *p* < 0.01 *	r = 0.38 *p* = 0.01
Total ghrelin (pg/mL)	r = −0.4 *p* = 0.03 *	r = 0.2 *p* = 0.1	r = 0.2 *p* = 0.1
Total energy intake (kcal)	r = 0.3 *p* = 0.08	r = −0.31 *p* = 0.06	r = 0.16 *p* = 0.35
Carbohydrate (g)	r = 0.26 *p* = 0.14	r = −0.26 *p* = 0.11	r = 0.18 *p* = 0.28
Protein (g)	r = 0.41 *p* = 0.02 *	r = −0.32 *p* = 0.04	r = 0.19 *p* = 0.25
Total fat (g)	r = 0.38 *p* = 0.03 *	r = −0.38 *p* = 0.02 *	r = 0.22 *p* = 0.18
SFA (g)	r = 0.26 *p* = 0.14	r = −0.51 *p* < 0.01 *	r = 0.34 *p* = 0.04 *
MUFA (g)	r = 0.35 *p* = 0.04 *	r = −0.35 *p* = 0.03 *	r = 0.19 *p* = 0.25
PUFA (g)	r = 0.34 *p* = 0.05	r = −0.28 *p* = 0.08	r = 0.14 *p* = 0.38
LC−PUFA (g)	r = 0.12 *p* = 0.49	r = −0.12 *p* = 0.46	r = 0.11 *p* = 0.52
Total dietary cholesterol (mg)	r = 0.15 *p* = 0.4	r = −0.13 *p* = 0.43	r = 0.06 *p* = 0.71
Percentage of energy from carbohydrate (%)	r = −0.04 *p* = 0.83	r = 0.05 *p* = 0.78	r = 0.05 *p* = 0.75
Percentage of energy from protein (%)	r = 0.01 *p* = 0.94	r = 0.16 *p* = 0.33	r = −0.18 *p* = 0.28
Percentage of energy from fat (%)	r = 0.22 *p* = 0.2	r = −0.24 *p* = 0.14	r = 0.11 *p* = 0.49

Values are expressed as the median (interquartile range): * *p* < 0.05. Abbreviations: SFA: saturated fatty acids; MUFA: monounsaturated fatty acids, PUFA: polyunsaturated fatty acids; LC-PUFA: long chain polyunsaturated fatty acids; PCOS: polycystic ovary syndrome; HOMA-IR: homeostasis model assessment of insulin resistance; SHBG: Serum sex hormone–binding globulin; FAI: free androgen index.
